# Supplementary material for: Two-Dimensional Large Gap Topological Insulators with Tunable Rashba Spin-Orbit Coupling in Group-IV films
Source: Sci Rep. 2017 Apr 3;7:45923. doi: 10.1038/srep45923 (PMC5377469; doi:10.1038/srep45923)
Supplement: Supplementary Material [file srep45923-s1.pdf]

Supplementary Information

Two-Dimensional Large Gap Topological Insulators with Tunable Rashba Spin-Orbit Coupling in Group-IV films

Shou-juan Zhang[a] , Wei-xiao Ji[a] , Chang-wen Zhang[a]*, Ping Li[a] & Pei-ji Wang [a]

[a] School of Physics and Technology, University of Jinan, Jinan, Shandong, 250022, People's Republic of China

Table SI Calculated equilibrium lattice parameters a (Å), the buckled height h(Å),band gap E_g (eV), band gap with SOC E_g (eV), the band gap located at Γ point E_Γ (eV), gap at Γ with SOC $E_{\Gamma\text{-SOC}}$ (eV) and topological Invariants (Z_2) for ABF_2 (A≠B= Si, Ge, Sn, Pb) systems.

**Structure**	***a***(Å)	***h***(Å)	E_g(eV)	E_Γ(eV)	$E_{g\text{-}SOC}$(eV)	$E_{\Gamma\text{-}SOC}$(eV)	Z_2
$PbSnF_2$	5.10	0.44	0	0	0.544	0.615	1
$PbGeF_2$	5.00	0.42	0	0	0.462	0.566	1
$PbSiF_2$	4.98	0.51	0	0	0.353	0.468	1
$SnGeF_2$	4.96	0.45	0	0	0.192	0.235	1
$SnSiF_2$	4.93	0.51	0	0	0.120	0.167	1
$GeSiF_2$	4.09	0.43	0	0	0.630	0.630	0

Table SII Calculated equilibrium lattice parameters a (Å), the buckled height h(Å),band gap E_g (eV), band gap with SOC E_g (eV), the band gap located at Γ point E_Γ (eV), gap at Γ with SOC $E_{\Gamma\text{-SOC}}$ (eV) and topological Invariants (Z_2) for $ABBr_2$ (A≠B= Si, Ge, Sn, Pb) systems.

**Structure**	***a***(Å)	***h***(Å)	E_g(eV)	E_Γ(eV)	$E_{g\text{-}SOC}$(eV)	$E_{\Gamma\text{-}SOC}$(eV)	Z_2
$PbSnBr_2$	5.08	0.62	0	0	0.263	0.519	1
$PbGeBr_2$	5.03	0.45	0	0	0.108	1.901	1
$PbSiBr_2$	5.05	0.19	0	0	0.150	0.306	1
$SnGeBr_2$	4.90	0.12	0	0	0.255	0.255	1
$SnSiBr_2$	4.58	0.62	0	0	0.120	0.203	1
$GeSiBr_2$	4.16	0.68	0.411	0.411	0.305	0.305	0

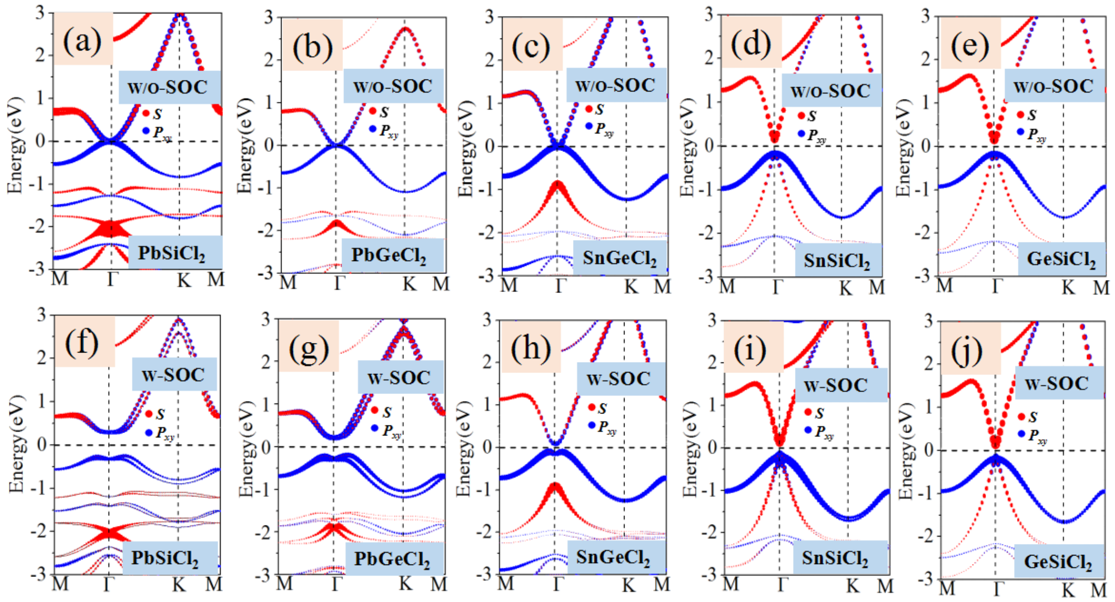

Fig. S1 Orbitals-resolved band structures without and with SOC for $ABCl_2$ (A, B=Si, Ge, Sn, Pb). The blue dots show *s* orbit composition and the red dots show $p_{x,y}$ orbit composition.

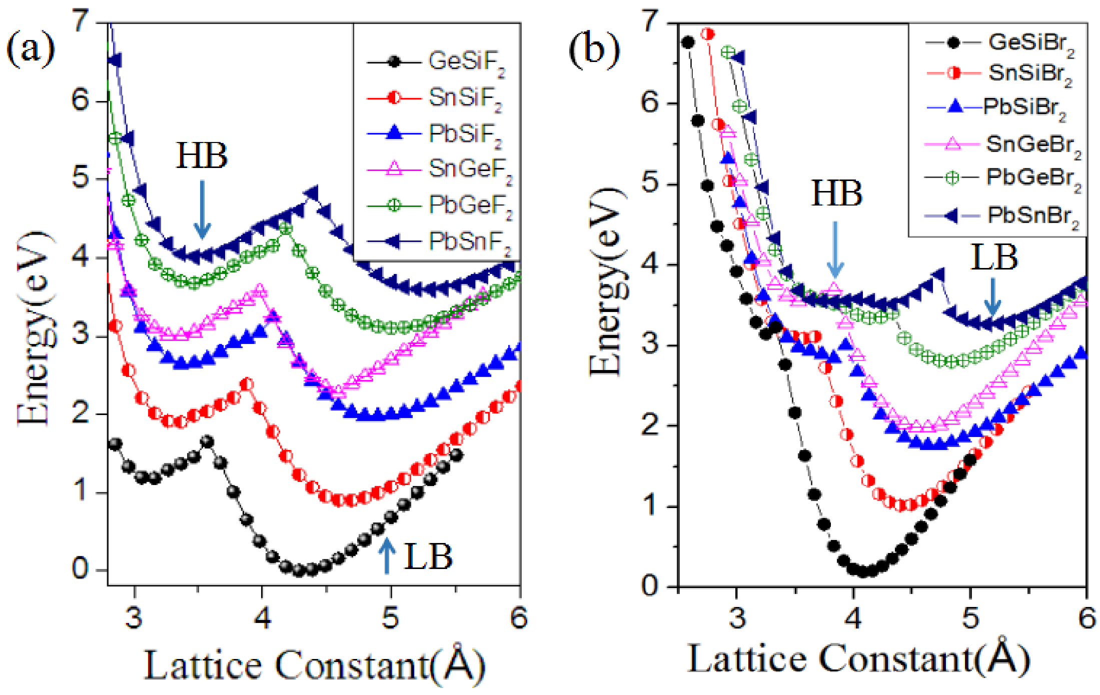

Fig. S2 Total energy of ABF_2 (A, B= Si, Ge, Sn, Pb) and $ABBr_2$ (A, B= Si, Ge, Sn, Pb) as a function of lattice constant, respectively.
